# Inorganic Interface Engineering for Stabilizing Zn Metal Anode

**DOI:** 10.1007/s40820-025-01922-x

**Published:** 2026-01-01

**Authors:** Shuguo Yuan, Wenqi Zhao, Zihao Song, Hai Lin, Xiangyang Zhao, Zhenxing Feng, Zhichuan J. Xu, Hongjin Fan, Qingli Zou

**Affiliations:** 1 State Key Laboratory of Chemical Resource Engineering, Beijing University of Chemical Technology, Beijing, 100029 People’s Republic of China; 2 School of Chemical, Biological, and Environmental Engineering, Oregon State University, Corvallis, OR 97331 USA; 3 School of Materials Science and Engineering, Nanyang Technological University, Singapore, 639798 Singapore; 4 School of Physical and Mathematical Sciences, Nanyang Technological University, Singapore, 637371 Singapore

**Keywords:** Zn metal batteries, Interface engineering, Aqueous electrolytes, Dendrite-free

## Abstract

Aqueous zinc (Zn) metal batteries (AZMBs) have distinct advantages in terms of safety and cost-effectiveness. However, the industrial application of AZMBs is currently not ready due to challenges of Zn dendrite growth and the side reactions such as hydrogen evolution reaction (HER) on the Zn anodes. In this review, we discuss how inorganic interfaces impact the Zn^2+^ plating/stripping reaction and overall cell performance. The discussion is categorized based on the types of inorganic materials, including metal oxides, other metal compounds, and inorganic salts. The proposed protection mechanisms for Zn metal anodes are highlighted, with a focus on the dendrite and HER inhibition mechanisms facilitated by various inorganic materials. We also provide our perspective on the rational design of advanced interfaces to enable highly reversible Zn^2+^ plating/stripping reactions toward highly stable AZMBs, paving the way for their practical implementation in energy storage.

## Highlights

- A broad overview of the inorganic interface engineering strategies, along with deep analysis of the mechanisms on regulating the Zn^2+^ plating/stripping process.
- Identify the limitations of interface engineering strategies and provide our perspective on the future research, highlighting more comprehensive analysis of the interfaces.

## Graphical abstract

**Figure:**
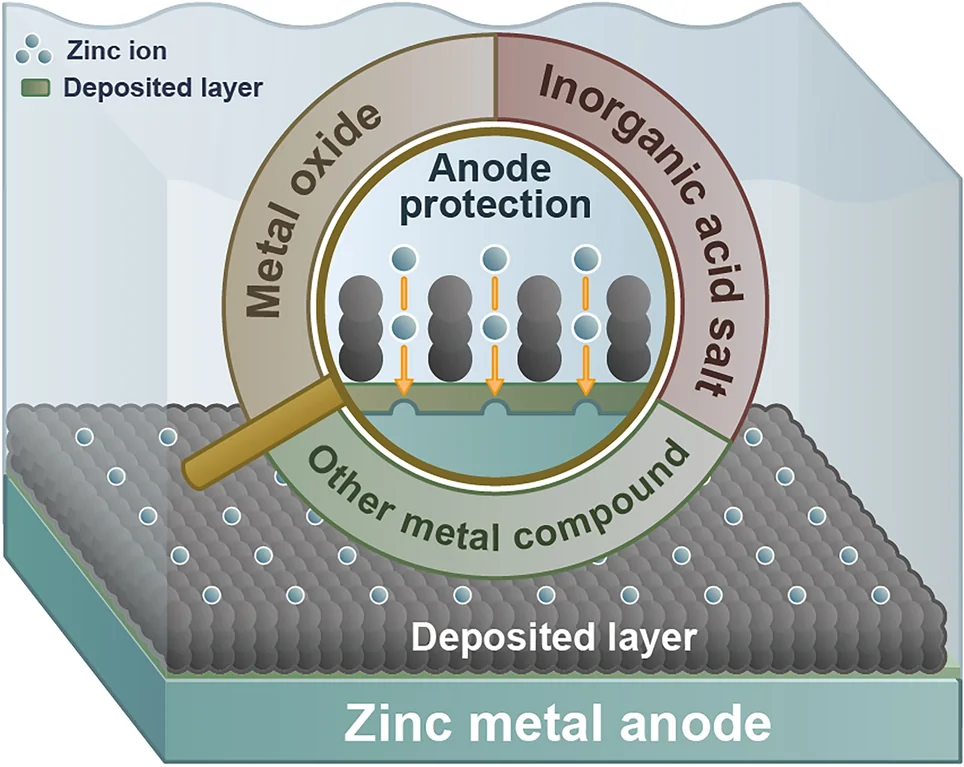

## Introduction

In recent years, the expansion of the global economy and increasing pollution issues have brought to the forefront escalating concerns about green energy, leading to an urgent demand for advanced energy storage systems [1, 2]. Lithium-ion batteries (LIBs), as the state-of-the-art energy storage technology, are unable to fully meet the demands for large-scale applications due to the safety issues associated with flammable organic electrolytes [3] and the limited resource availability of lithium [4].

**Fig. 1:**
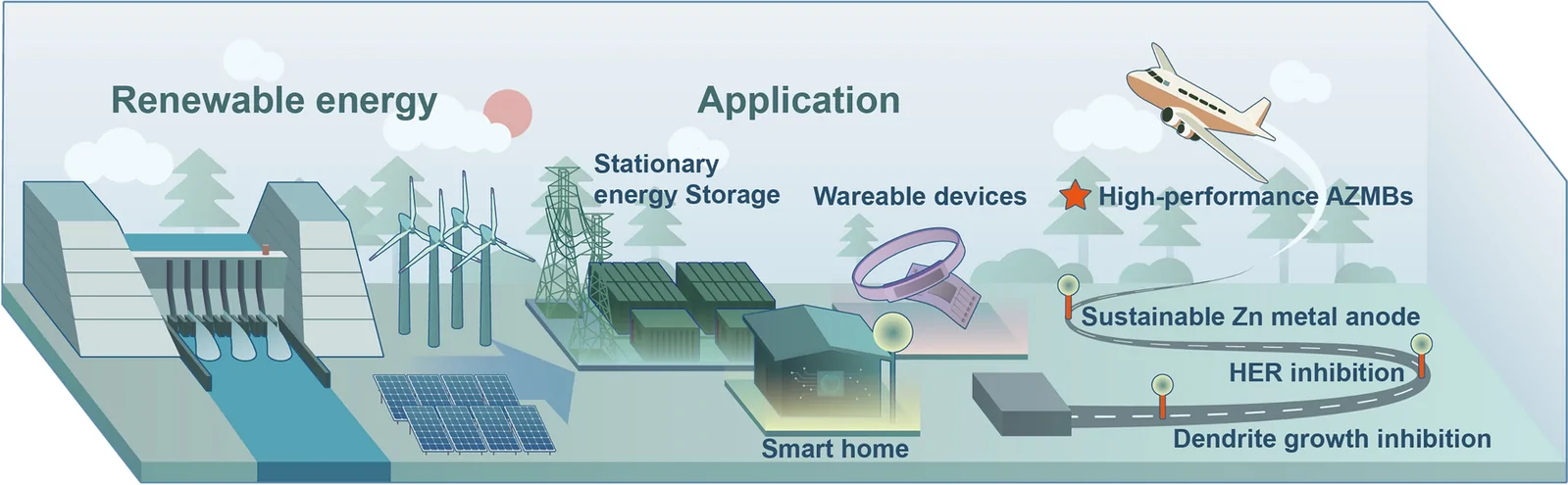
Application scenarios and future development trend of aqueous Zn metal batteries

Aqueous Zn metal batteries (AZMBs) have garnered increasing attention due to their obvious advantages in terms of safety and cost (Fig. 1). Zn metal, with its high natural abundance, shows a much lower price compared to lithium. Additionally, the use of nonflammable aqueous electrolytes greatly eliminates safety concerns [5]. Furthermore, the fast ion-transport nature of aqueous electrolytes allows for superior power output, making AZMBs promising for large-scale applications [6–8]. However, the industrial application of AZMBs is currently restricted due to the severe issues of dendrite growth and hydrogen evolution reaction (HER) on Zn metal anodes [9, 10]. The uneven electric field distribution at the anode-electrolyte interface leads to the uneven plating of Zn ions [11], further resulting in the formation of sharp dendrites that can cause short circuits and limit the cycling life. Moreover, the HER, as a side reaction occurring at the anode-electrolyte interface, leads to the consumption of the electrolyte and passivation of the Zn metal anode [12], further limiting the cycling life of AZMBs.

To address these issues and achieve high-performance AZMBs, considerable efforts have been devoted to the structural and compositional design of Zn metal anode [13], electrolyte [14], and anode-electrolyte interface [15]. Among the various approaches, interface engineering strategies have emerged as particularly promising for effectively inhibiting Zn dendrite growth and mitigating side reactions [16]. These strategies involve either modifying the surface of the original Zn foil or constructing entirely new functional interfaces composed of organic, inorganic, or composite materials [17, 18]. By promoting uniform ion conduction and charge distribution, these strategies enable uniform and dendrite-free Zn deposition [19]. Furthermore, by isolating the direct contact between water and Zn anode, the HER can be effectively inhibited [20]. Notably, interface engineering strategies using inorganic materials offer the additional benefits of high rigidity and corrosion resistance [21], which can further reduce the risk of short circuits caused by dendrite puncture and chemical corrosion at the interface.

**Fig. 2:**
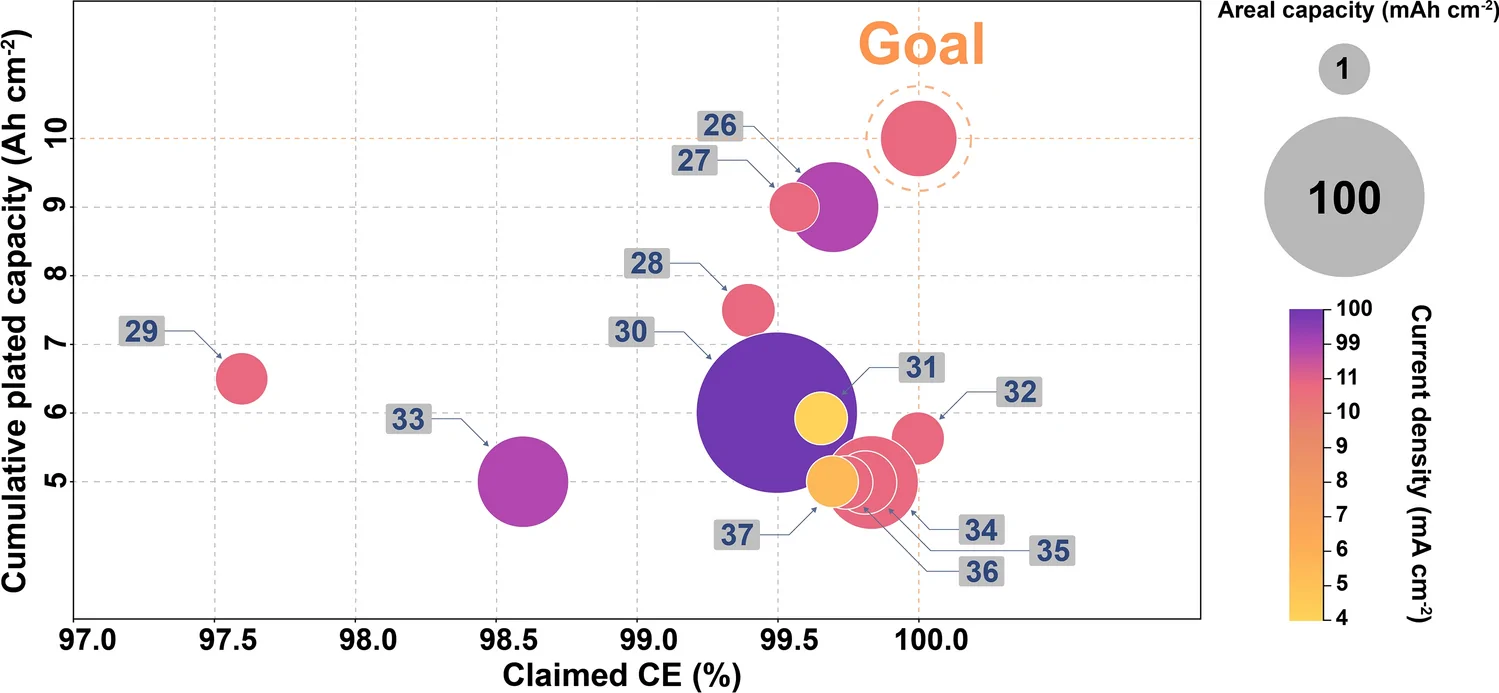
A literature survey of Zn plating/stripping properties, including Coulomb efficiency (CE), cumulative plated capacity, areal capacity, and current density. The cumulative capacity is the summation of the capacity plated on each individual cycle and can be obtained by multiplying the capacity plated per cycle and the number of cycles [26–37]

Beyond these basic functions, the interface engineering strategies, especially those based on inorganic materials, show great application prospects [22] and potential for large-scale energy storage. This is due to their additional advantages, such as low material cost, facile preparation process, and tolerance for high areal capacities [23]. With no restrictions on the choice of raw electrode substrates, this strategy can leverage commercially available Zn metal foils-the most promising anode material for AZMBs to maximize economic efficiency [24]. The inorganic modification layers themselves are also inherently inexpensive. More importantly, the modified interfaces based on inorganic materials exhibit non-consumable properties. Unlike some electrolyte additives, these inorganic interfaces generally do not need to participate in reactions as reactants to provide functionality [22]. Due to their high chemical and electrochemical inertness, they are less likely to cause side reactions [25]. This non-consumable and inert nature of the inorganic interfaces support the realization of practical-level high surface capacities and long cycle life. Based on this approach, researchers have achieved surface capacities close to practical targets (Fig. 2).

In this review, we present the progress made in interface engineering strategies based on inorganic materials for AZMBs. We discuss the influence of interface properties on the Zn^2+^ plating/stripping reaction and overall cell performance. The materials are classified as metal oxide materials, other metal compound materials, and inorganic salt layers. Our intention is to highlight the proposed protection mechanisms for Zn metal anodes, with a focus on mechanisms of inhibiting dendrite growth and HER enabled by various inorganic materials. Finally, we provide conclusions and perspectives on the design of advanced Zn^2+^ plating/stripping reaction interfaces to achieve high-performance AZMBs, especially in terms of meeting practical requirement.

## Challenges Faced by Zn Metal Anodes

Despite the advantageous features of Zn anodes, such as non-toxicity, high energy density [38], and low cost, their commercial applications still remain limited due to the low Coulombic efficiency and restricted cycling life, which originate from the two major issues: dendrite growth and HER [39].

The formation of dendrites is mainly attributed to the uneven charge distribution and ion diffusion at the anode-electrolyte interface [40]. Uneven charge distribution on the electrode surface leads to the initial uneven nucleation during plating. The uneven diffusion of Zn ions promotes the growth of Zn crystal nuclei toward the bulk electrolyte, where the Zn ion concentration is higher [41]. In neutral or acidic electrolytes, Zn^2+^ gains electrons at the anode-electrolyte interface during plating to form Zn, which exhibits a two-dimensional hexagonal crystal structure [42, 43]. In alkaline electrolytes, it is ZnO that gains electrons to form Zn. Although the Zn anode reacts differently in various electrolyte systems, the crystal structure all display sharp tips, which will further exacerbate the growth of dendrites due to the “tip effect” [44]. When reaching a critical point, these dendrites may puncture the separator, causing short circuits, ultimately limiting the cycling life [45]. According to Sand's time model, the rate of ion migration and current density will also affect the growth of dendrites, which is described as follows:

1 $$\tau =\pi D\frac{\varepsilon {C}_{0}{\left({\mu }_{a}+{\mu }_{c}\right)}^{2}}{4J{\mu }_{a}}$$

where τ is Sand's time, in AZMBs, the time at which the Zn^2+^ concentration at the electrode surface drops to zero-originally introduced as Sand’s time-has been extended to characterize the onset of zinc dendrite formation. First employed by Sand in 1899 to study copper dendrite growth in aqueous electrolytes, the concept has been widely adopted across diverse electrolyte systems over the past decade [46]. And the smaller it is, the easier it is to generate dendrites. *D* is the ion diffusion constant, *C*_0_ is the initial concentration of the reactive ions, *J* is the effective current density, and *μ*_a_ and *μ*_c_ are the mobility of anions and cations, respectively. It can be seen from the above equation that dendrite growth will be more serious at high current density. Tao et al. [47] proposed that at high current densities, turbulence occurs at the electrode interface, significantly affecting the homogeneous deposition of Zn^2+^. During cycling, this interfacial turbulence can accelerate Zn dendrite growth by disrupting ion transport. Implementing interface engineering strategies to effectively mitigate this turbulence could help achieve high surface capacity in aqueous zinc batteries. However, other studies have demonstrated a contrasting result, showing that increased current density leads to improvements in both reversibility and cycling time [48, 49]. At the same areal capacity, increasing the current density from 0.033 to 20.0 mA cm^-2^ led to a significant improvement in coulombic efficiency, from 56.2% to 97.3% [50]. Cycling tests have provided consistent results: As the current density increased from 1 to 10 mA cm^-2^, the cycling Life of Zn-Ti cells showed over a sixfold increase, from approximately 150 to 1000 h. This phenomenon is attributed to inhibited dendrite formation under high current conditions, which promotes the formation of nuclei, resulting in denser and more uniform deposition. These findings also illustrate the significant impact of dendrite growth on the coulombic efficiency and cycle life of batteries.

The occurrence of the HER at the anode-electrolyte interface can be attributed to its much lower reaction potential compared to the main reaction of the Zn anode in all electrolyte systems [51]. Current research primarily focuses on mild acidic electrolyte systems, and the standard electrode potential of Zn^2+^/Zn [-0.76 V vs. standard hydrogen electrode (SHE)] is lower than that of H^+^/H_2_ (0 V vs. SHE) [51]. Similarly, in alkaline electrolytes, the standard redox potential of Zn/ZnO (-1.26 V vs. SHE) is also lower than that of H_2_O/H_2_ (-0.83 V vs. SHE) [52]. This means that the HER reaction is thermodynamically favored, regardless of the pH conditions. Although HER exhibits slow reaction kinetics on the Zn metal surface, the cumulative consumption of electrolytes by HER cannot be ignored as the charge-discharge cycles proceeds. This parasitic HER significantly affects the Coulombic efficiency and cycle life of the battery [53].

It is crucial to note that there is a mutually exacerbating correlation between dendrite growth and HER [54]. The presence of Zn dendrites can aggravate HER and other side reactions, due to the enlarged contact area between the plated Zn anode and the electrolyte, resulting in the depletion of the Zn anode and electrolyte [46, 47]. The HER reaction will occupy a portion of the reaction sites on the surface of the Zn anode, while also causing an increase in the local OH^-^ concentration [11]. This can further trigger other side reactions to form insoluble by-products. These insoluble deposits will passivate the Zn metal anode and exacerbate the unevenness of the electrode surface, leading to even more severe dendrite growth issues [55].

Therefore, regulating the stability of the Zn metal anode and electrolyte interface in AZMBs is essential to suppressing Zn dendrites, corrosion, HER, and ensuring the safety and stability of battery systems.

## Inorganic Materials

**Fig. 3:**
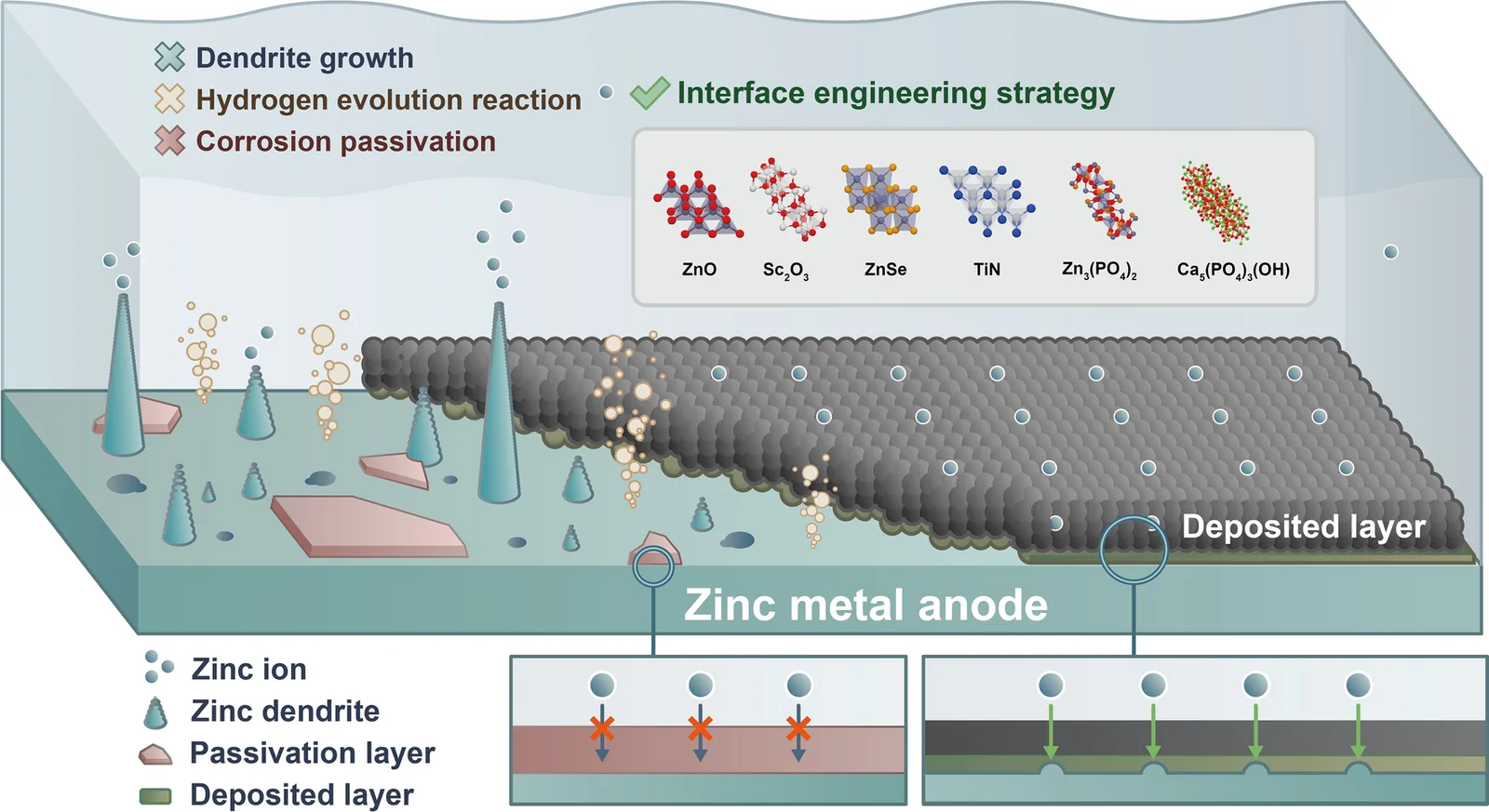
Schematic diagram of interactions with aqueous electrolyte on the Zn metal anode surface with or without inorganic layer

Interface engineering strategies based on inorganic materials hold great promise for constructing high-stability, dendrite-free Zn anodes [11], as shown in the following (Fig. 3). The inorganic materials used for preparing interface protection layers should meet some basic requirements, including good electrochemical/chemical stability, excellent ionic conductivity, cost-effective, and controllable production. Owing to the polar inorganic layers constructed between the Zn anode and the electrolyte, the charge distribution can be regulated on the electrode surface and the diffusion of Zn ions can be simultaneously controlled through the adsorption of Zn ions, thereby achieving uniform Zn deposition [56]. The dense inorganic interface layer can also isolate the direct contact between water molecules and conductive Zn metal anodes, thereby inhibiting the HER [57, 58]. On this basis, different inorganic materials have exhibited distinct regulating mechanisms for Zn metal anodes, depending on the specific types and characteristics of the applied materials. Therefore, we will systematically discuss the specific regulating mechanism and electrochemical performance of Zn metal anode with inorganic interface layers based on metal oxides, other metal compounds, and metal salts.

### Metal Oxides

Metal oxides are known for their robust chemical and electrochemical stability, which facilitates the achievement of long-term cycling. Moreover, metal oxides usually have high dielectric constants and polarity, undergoing polarization during charging and discharging processes. This results in the accumulation of positive charges at the interface between Zn anodes and metal oxide layers, while the opposite end is rich in negative charges [59]. Thus, the metal oxide layers can homogenize the charge distribution on the Zn anode and enable uniform deposition of Zn^2+^, while their high mechanical strength effectively suppress the formation of Zn dendrites. In addition, the compact structure of metal oxide layers effectively isolates direct contact between the Zn anode and the electrolyte, significantly restraining the formation of hydrogen gas and by-products during cycling [23]. For instance, a porous rutile nano-TiO_2_ layer on Zn metal surface is proven effective in guiding uniform deposition of zinc ions and inhibiting the formation of zinc dendrite [56]. At the current density of 0.4 mA cm^-2^, the cycle Life is increased by about 40 h and the overpotential is reduced by about 50 mV compared with bare Zn. Furthermore, in a similar report by Song et al. [60], a TiO_2_/NCDs hybrid layer was employed, in which the TiO₂ layer prevents direct contact with water molecules, and the NCDs (N-doped carbon dots) layer, which are rich in functional groups (-OH, -COOH, -NH₂), provide zincophilic nucleation sites. These sites enable a low deposition overpotential of 28 mV and guide the Zn deposition into a petal-like structure.

Zinc oxide [32, 61] is another widely utilized interface modification material. For example, as demonstrated by Zhou et al. [62] a ZnO coating by in situ Zn(OH)_4_^2-^ deposition results in accelerated kinetics of Zn^2+^ transfer and deposition via electrostatic attraction between the O sites of ZnO and Zn^2+^. As a result, the modified anode achieves an average Zn utilization of 99.55% and demonstrates long-term stability for 1000 cycles at a current density of 5 mA cm^-2^. Meanwhile, Zn-MnO_2_ cells incorporating this modified electrode exhibit negligible capacity fading after 500 cycles at 0.5 A g^-1^, Maintaining a specific capacity of 212.9 mAh g^−1^. Other metal oxide layers reported include Al_2_O_3_ [63], Sc_2_O_3_ [64], Nb_2_O_5_ [65], and ZrO_2_ [66].

#### Design of Crystal Structure

**Fig. 4:**
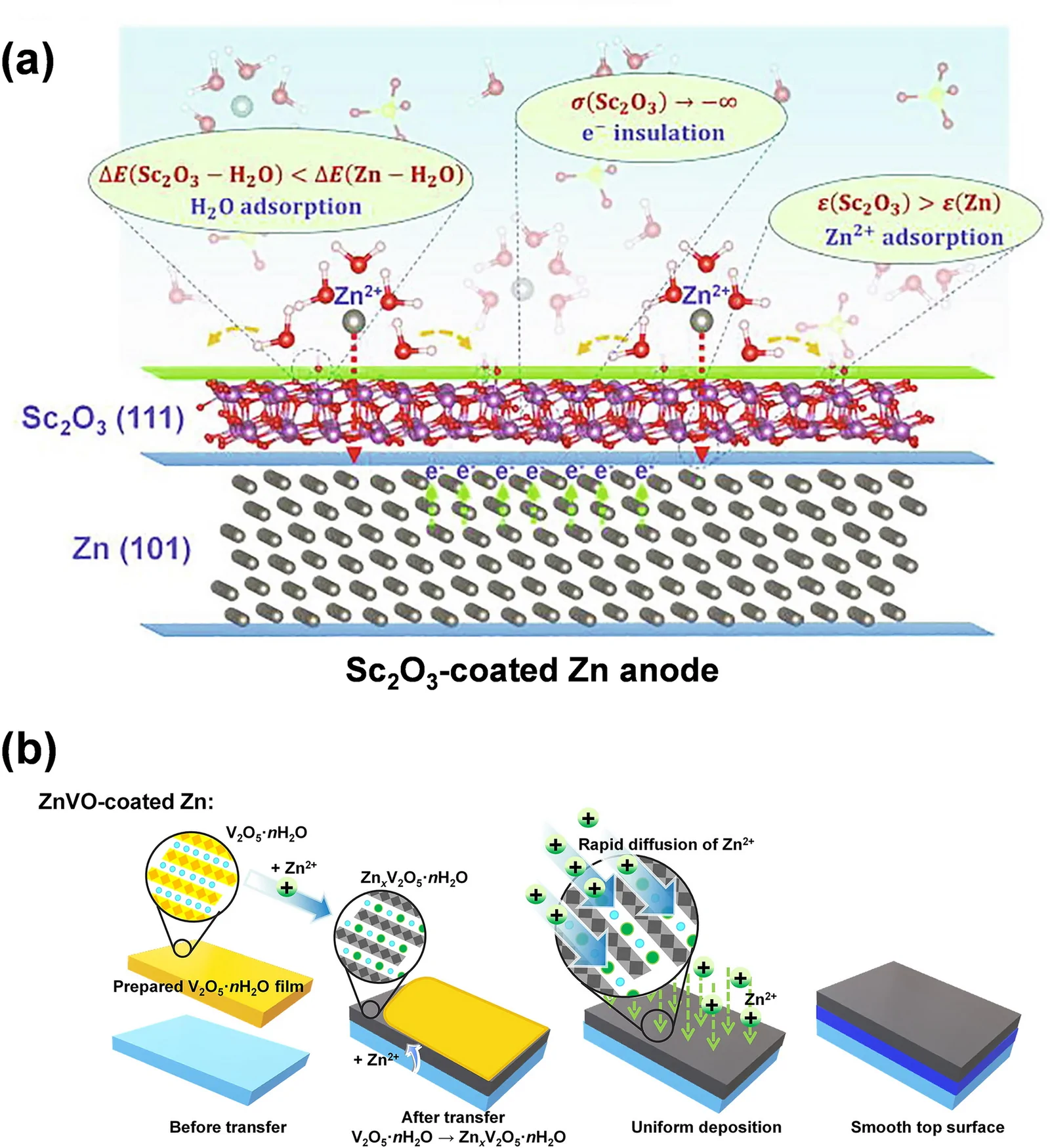
Metal oxide layers regulating zinc anode surface deposition. **a** Schematic diagram of the reaction process on the surface of the Sc_2_O_3_-coated Zn anode [64]. Copyright 2021, Elsevier. **b** Schematic diagram and mechanism of the morphology evolution of ZnVO-coated Zn [67]. Copyright 2021, Elsevier

Building upon the concept of uniform surface charge distribution, the crystal structure of the metal oxides plays an important role in determining the ionic conduction rate, which in turn affects the maximum achievable current for AZMBs employing this strategy. Some oxide crystal structures possess channel-like features that can facilitate rapid conduction of Zn ions, leading to high power performance. Liang et al. [64] constructed an artificial Sc_2_O_3_ layer with channel structures oriented along the (111) crystal plane (Fig. 4a). The Sc_2_O_3_ coating was made of many small particles with multichannel structure, which makes it beneficial to the diffusion of Zn^2+^ ions and the generation of uniform charge distribution. Benefiting from this oriented channel structure, the Sc_2_O_3_-coated Zn anode can run for more than 100 cycles without short circuit and exhibit low voltage hysteresis.

This Zn surface protection strategy can also extend to composite oxide protective layers. Zhai et al. [67] show that an in situ formed Zn_x_V_2_O_5_-nH_2_O layered (Fig. 4b) on the Zn metal surface provides channels for rapid Zn^2+^ insertion and promote uniform deposition. And the lattice spacing of (001) plane is 11.30 Å in V_2_O_5_·1.6H_2_O, which is large enough for the insertion/extraction of zinc ions. At a current density of 0.25 mA cm^-2^ and 0.05 mAh cm^-2^, stable cycling at a lower overpotential was achieved for over 560 h, and in Full cells, 1000 stable cycles were Maintained with a Coulombic efficiency of 99%. Yang et al. [68] proposed an Mg-Al layered double hydroxide (LDH)-based artificial SEI with Zn ion channels. The Mg^2+^ and Al^3+^ cations are located at the center of the octahedron, and hydroxide anions occupy the vertexes which were connected to form a 2D infinite layer structure. Mg-Al LDH has two-layer structures: type I (7.70 Å) and type II (7.36 Å), both large enough for hydrated Zn^2+^ ions (~5.5 Å), and facilitates Zn^2+^ rapid transport. These ion channels in Mg-Al LDH can effectively redistribute Zn ion flux, leading to stable Zn plating/stripping underneath the artificial SEI.

#### Design of Interface Structure

Although the metal oxide layers can improve the performance of the Zn metal anode to certain degree, it is still far from being sufficient for practical application. To better address this, researchers have explored various structural designs for the interface, including nanostructures, concave structures, pyramid array structures, hollow structures, etc. These structured interlayers have proved more effective in further elevating the performance of AZMBs, even under harsh operating conditions such as high current densities and wide temperature ranges.

Atomic layer deposition method has been deployed in AZMBs to deposit nanoscale oxide thin films (< 10 nm) with high uniformity in thickness. For example, Liu et al. [63] deposited nanoscale Al_2_O_3_ layer (∼10 nm) on Zn metal anode, resulting in a stronger mechanical strength, uniform Zn^2+^ concentration on the electrode surface, and suppressed dendrite formation. Additionally, it reduces the reactivity between the Zn anode and the electrolyte, suppressing side reactions and the formation of Zn(OH)_2_. This atomic thick coating allows the Zn-Zn symmetric cell to stably cycle over 500 h at the current density of 1 mA cm^-2^.

**Fig. 5:**
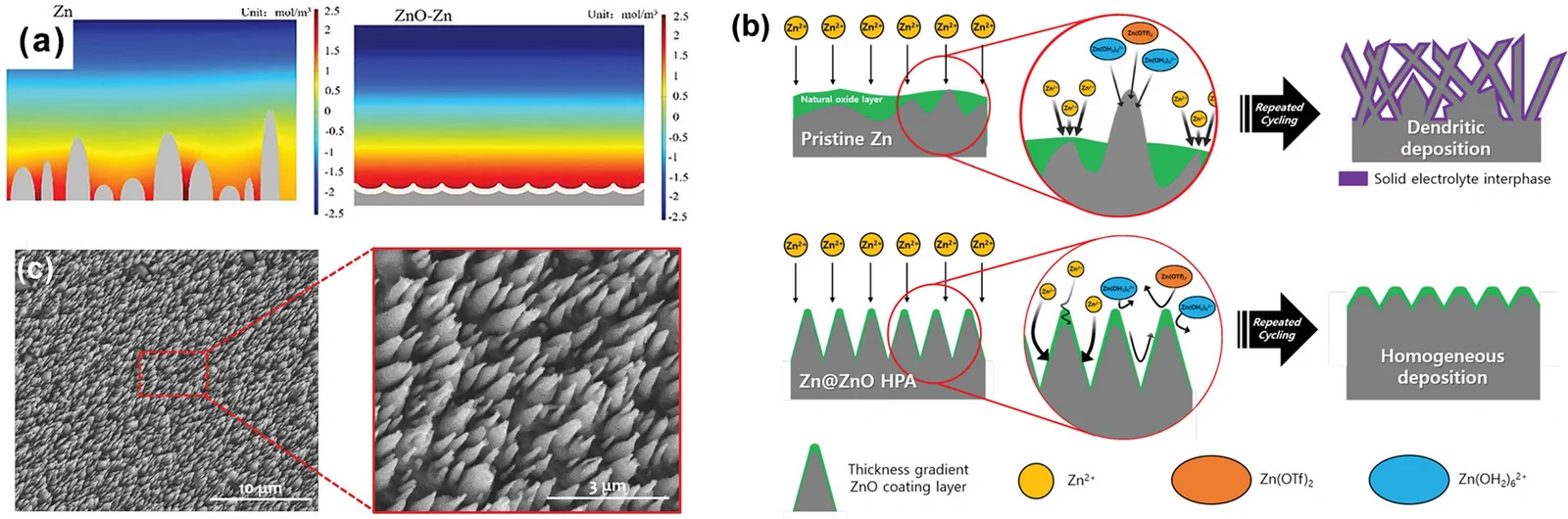
Design of interface structure. **a** Simulated Zn ion concentration field models based on Zn and ZnO-Zn anodes [32]. Copyright 2023, Wiley‐VCH. **b** Schematic diagram of the deposition behavior of Zn ions in aqueous solution on pure Zn anode and Zn@ZnO hexagonal pyramid array anode. **c** Top-view SEM image of the hexagonal pyramid array [61]. Copyright 2020, Wiley-VCH

In addition to atomic-thin layers, microscopic convex or concave structures also can provide a regulatory effect on the charge distribution at the anode–electrolyte interface. For example, a ZnO layer on the Zn metal anode with a micro-concave structure is found effective in promoting a uniform electric field distribution and ion migration (Fig. 5a) [32]. The concave shape aids in facilitating the flow of zinc ions within the Material, enabling stable cycling for 3800 cycles at 50 mA cm^-2^. In a similar work by Lee et al. [61], a micro-convex structure of densely distributed Zn hexagonal pyramid arrays covered with a thin gradient ZnO layer was constructed (Fig. 5b, c). The gradient thickness ZnO layer had lower resistance at the sides and bottom of the pyramids, leading Zn^2+^ to preferentially deposit there, enabling uniform plating. This gradient layer also suppresses HER as it blocks direct contact with the electrolyte, accounting for a tenfold increase in cycle life compared to bare Zn anode.

Another type of interface structuring is the hollow structure featuring a thin shell and inner void that might benefit electrolyte contact and ion diffusion. For example, it has been shown that the hollow architecture of the protective ZnSnO_3_ cubes (HZTO) layer facilitates Zn^2+^ diffusion, accounting for the observed high charge transfer kinetics and plating/stripping reactivity [13].

### Other Metal Compound Materials

In addition to the above discussed metal oxides, various other metal compounds have also been extensively utilized as the protective layers for Zn metal anodes, exemplified by nitrides [69, 70], selenides [71, 72], sulfides [73], and MXene that are to be elaborated in the following.

#### Metal Nitrides, Selenides, and Sulfides

As N, Se, and S atoms usually exhibit strong zincophilicity and high adsorption capacity for Zn^2+^ ions, and they will provide rich nucleation sites and facilitate uniform and dendrite-free deposition of Zn [72].

**Fig. 6:**
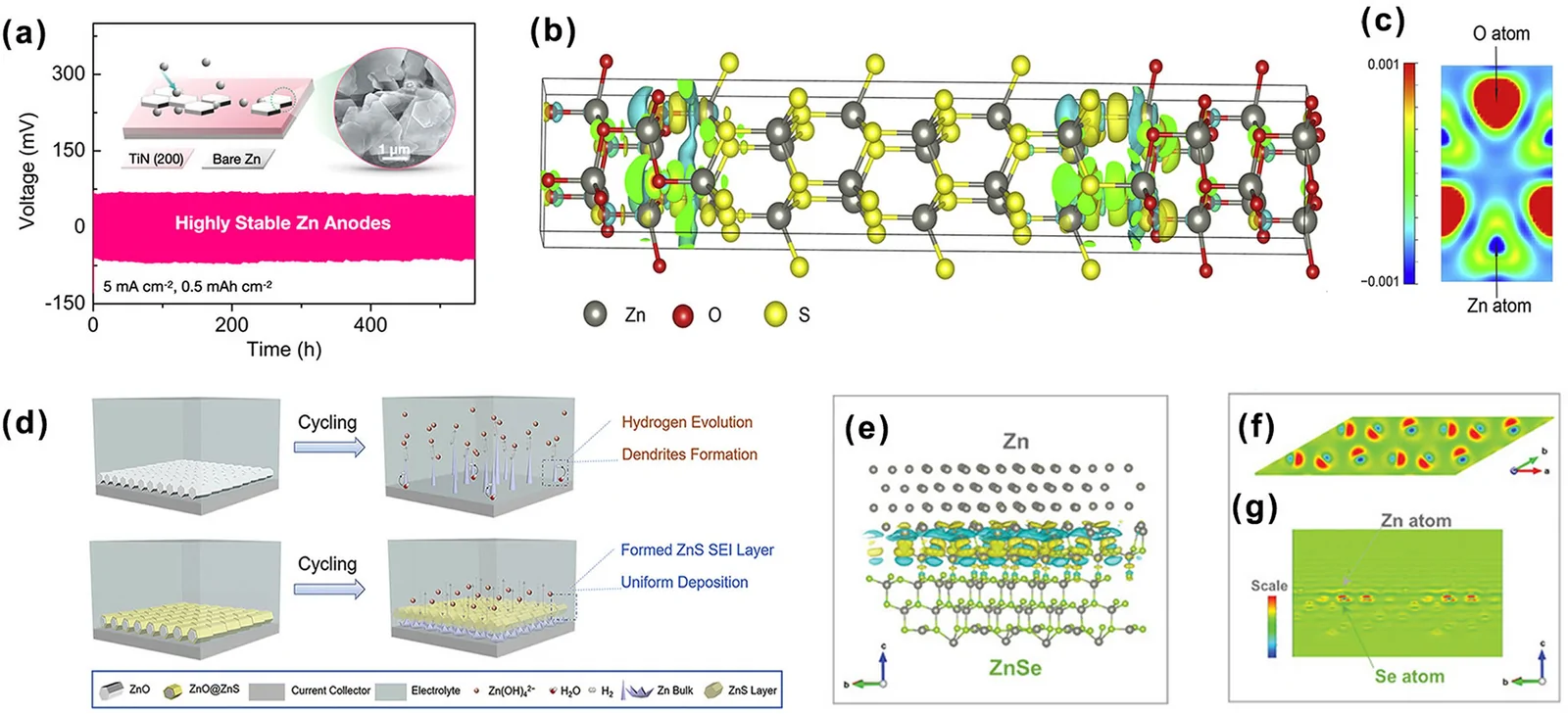
Zn deposition and charge distribution on various interfaces **a** Simulated deposition behavior of Zn(OH)_2_ on TiN layers [70]. Copyright 2023, The Royal Society of Chemistry. **b** Charge density difference distribution of the ZnO@ZnS [74]. **c** Sliced 2D contour map of the ZnO-ZnS interface [74]. **d** Schematic illustration of Zn deposition for pure ZnO and ZnO@ZnS_350_ anodes [74]. Copyright 2021, Wiley‐VCH. **e** Schematic illustration of the interface simulated by combining Zn (001) and ZnSe (111) surfaces and the electron density difference map at the interface [75]. **f** Cross section of the electron density difference map [75]. **g** Longitudinal section of the electron density difference map [75]. Copyright 2021, Elsevier

In recent years, there are a number of reports on the application of nitrides for interface modification of Zn metal anode. For example, CrN [69] has a higher adsorption capacity for Zn atoms than bare Zn, which provides abundant nucleation sites and induces uniform Zn deposition, thus preventing the growth of Zn dendrites. This beneficial effect is also observed from magnetron-sputtered TiN (200) thin layer (Fig. 6a) [70]. The TiN thin layer shows a strong orientation-dependent property; compared to other orientations, the (002) surface can inhibit the HER side reaction and, more interestingly, induce a lateral growth of by-product compared to the vertical growth on TiN (111) layer. Despite these advantages, the complex fabrication process and elevated production costs necessitate further research development.

In addition to nitrides, interface engineering strategies based on sulfides and selenides have also been reported for Zn anodes. Researchers have identified the presence of bonding interactions between the selenium or sulfur atoms and the Zn atoms at the interface between the Zn metal anode and the protective layer. This interfacial interaction alters the charge distribution, resulting in a vertical electric field, which accelerates the migration of Zn ions toward the negative electrode and enables fast Zn deposition and nucleation in both acidic and alkaline electrolyte systems. Two examples are provided below.

In the alkaline Zn metal battery system, Liu et al. [74] created a dense and uniform ZnS layer in situ on the ZnO anode (ZnO layer on Zn metal substrate) to enhance its stability. The ZnS@ZnO heterojunction promotes uniform Zn deposition by anchoring Zn(OH)_4_^2-^ ions as revealed by DFT calculations which reveals a strong adsorption energy of -3.40 eV due to unbalanced charge distribution at the ZnO and ZnS heterointerface. Moreover, strong interactions between S atoms and Zn atoms at the ZnO@ZnS interface, driven by charge migration and redistribution (Fig. 6b, c), establishes an unbalanced electric field that accelerates Zn^2+^ migration and effectively inhibits Zn dendrite formation (Fig. 6d). The ZnO@ZnS symmetric cells achieved stable battery cycling for over 1000 hours at a current of 17 mA cm^-2^.

In the acidic Zn metal battery system, Zhang et al. [75] presented a multifunctional protective layer comprising uniformly distributed and tightly packed ZnSe nanoparticles with sizes ranging from 100 to 200 nm. This dense layered structure uniformly covers the Zn foil surface, with a thickness of approximately 0.8 μm. Similar as the above case, the interaction between Se and Zn atoms at the interface alters the charge distribution, resulting in an imbalanced vertical charge distribution (Fig. 6e, g). This local internal driving force accelerates Zn^2+^ migration to the negative electrode, enabling fast and uniform deposition of Zn^2+^ beneath the protective layer, effectively inhibiting dendritic growth.

#### MXene

Two-dimensional transition metal carbides, known as MXenes, have emerged as promising materials for Zn anode protection, attracting significant research interest in recent years [22]. The general formula is M_n+1_X_n_T_x_, where M represents a transition metal, X is either C or N, and T denotes the surface end group. MXene materials boast excellent electronic conductivity, stable mechanical properties, diverse structures, and a hexagonal crystal system that aligns with Zn. This alignment enables swift electrochemical kinetics and uniform Zn deposition, leading to widespread exploration in the realm of Zn anode layer materials [36]. Niu et al. [76] applied a layer of MXene coating to the surface of the Zn anode, facilitating the uniform transmission of Zn^2+^ and electric field distribution, thereby achieving the suppression of Zn dendrites.

**Fig. 7:**
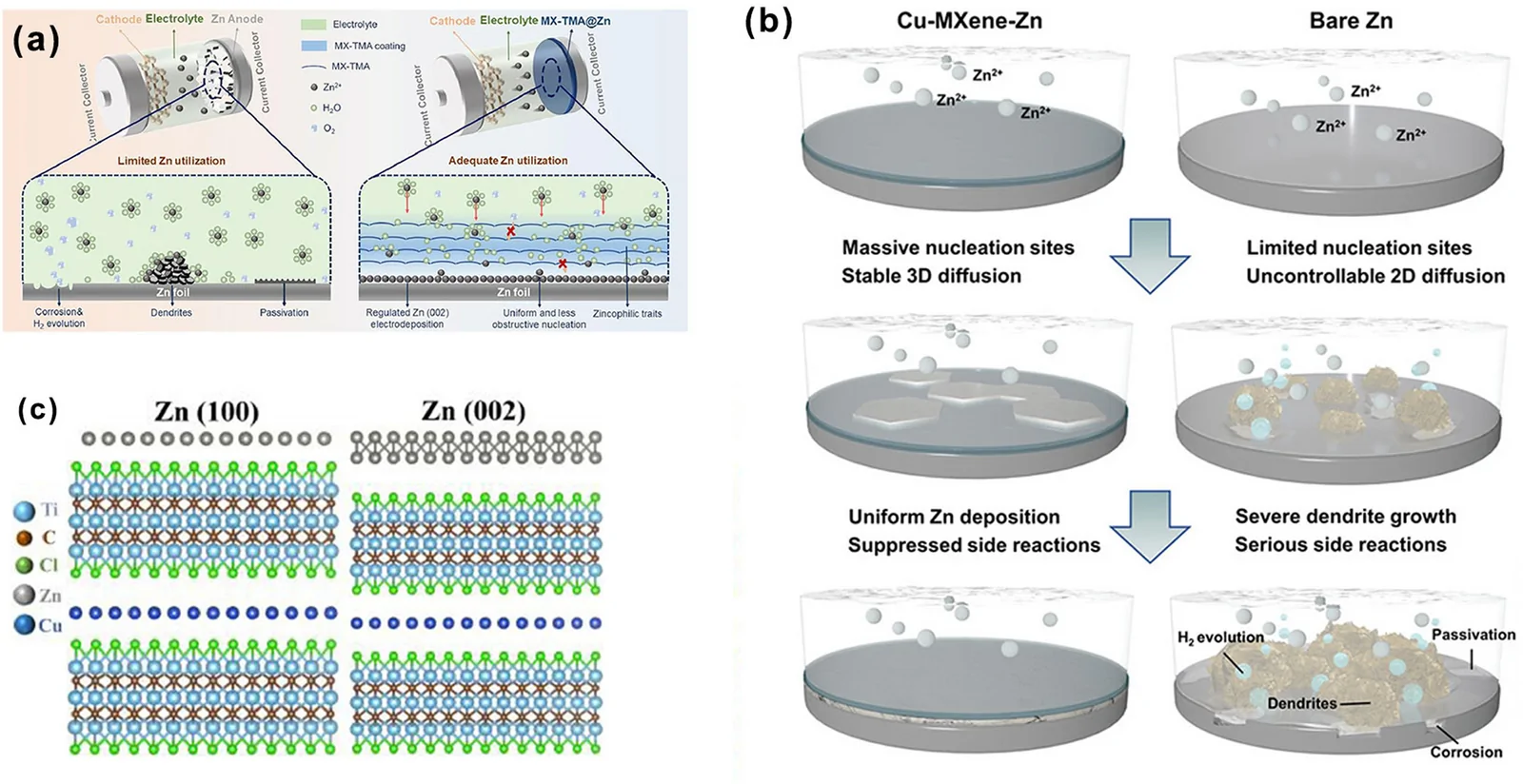
Zn deposition behavior on MXene layers.** a** Schematic diagram of the deposition behavior of Zn on bare Zn and MX-TMA@Zn [80]. Copyright 2020, The Royal Society of Chemistry. **b** Schematic diagram of the deposition of Zn on the surface of bare Zn and Cu-MXene-Zn [36]. Copyright 2021, Wiley-VCH. **c** Adsorption model of Zn^2+^ in CMX [81]. Copyright 2024, Elsevier

The modifications to Mxene were further investigated to enhance Zn^2+^ adsorption and hydrophobicity, targeting the uniform Zn deposition and inhibition of the hydrogen evolution side reaction. The suppression of the HER by MXene mainly relies on the following aspects: MXene can serve as a physical barrier that diminishes contact between water molecules and the zinc anode to suppress the HER [77]; on the other hand, surface functional groups on MXene can modulate the interfacial hydrogen-bond network, disrupt its continuity and hinder proton transport to prevent H₂ formation [78]; moreover, introducing specific functional groups (e.g., halogens or sulfur) can raise the energy barrier for HER, further enhancing the ability of MXene to inhibit hydrogen evolution [79]. Shen et al. [80], for instance, utilized a simple self-assembly method to prepare tetramethylammonium-modified Ti_3_C_2_T_x_ MXene (MX-TMA) material (Fig. 7a). Introducing numerous nitrogen-containing groups on the MXene surface, they established strong interactions with Zn^2+^, promoting the uniform deposition of Zn^2+^ and preventing the formation of Zn dendrites. The resulting Zn symmetric battery exhibited stable cycling for over 3600 h at 2 mA cm^-2^, with an average Coulombic efficiency of 99.75%. In terms of hydrophobic regulation, Xu et al. [36] employed a one-step molten salt etching method to produce Ti_3_C_2_Cl_2_ MXene layers with both high Zn affinity and hydrophobicity (Fig. 7b), leading to further suppression of side reactions. This layer, obtained by etching with CuCl_2_, incorporates numerous Cl functional groups, exhibiting excellent adsorption and hydrophobic properties for Zn^2+^. Even at high current densities of 10 mA cm^-2^ and 1 mAh cm^-2^, the battery can stably cycle for 1000 hours, Maintaining a Coulombic efficiency of 99.6%. Similarly, Chen et al. [81] constructed Cu/Ti_3_C_2_Cl_2_ MXene (CMX) materials with high Zn affinity and hydrophobicity. The CMX model features a layer of Cu sandwiched between two MXene layers as the substrate (Fig. 7c). Subsequent calculations of zinc adsorption energy on different substrate planes revealed that the (002) plane of the CMX composite had a lower adsorption energy (-0.51 eV) compared to the (100) plane (-0.74 eV), indicating that Zn^2+^ from the electrolyte is most likely to deposit preferentially along the (002) plane on CMX substrates. Therefore, it can maintain the horizontal growth of the Zn (002) plane During the subsequent cycles, thus inhibiting Zn dendrites. batteries coated with this protective layer show stable cycling for over 1400 h at 0.5 mA cm^-2^. The thickness of the MXene layer is considered a critical factor affecting both ionic conductivity and flexibility of the electrode. The MXene layer is electronically conductive and mechanically flexible. This uniqueness makes MXene thin layer versatile for Zn anode. For example, a thin (≈10 nm) Ti_3_C_2_T_x_ MXene layer was obtained by Liu et al. via the Marangoni-driven self-assembly and transferred onto the Zn foil surface [82]. This thin and soft MXene layer not only guides Zn directional deposition on the (002) crystal surface, but also obtains higher cyclic stability for the Zn anode due to the stress adaptation, showing a stable capacity in the soft package Zn-I_2_ battery over 1500 cycles at 1 A g^-1^. Although MXene materials can effectively inhibit the growth of metal anode dendrites, the scheme is tedious, and a simple and feasible scheme is still to be found.

### Inorganic Acid Salt Materials

In addition to the above-mentioned metal oxides and other metal compounds, some inorganic acid layers: phosphates, silicates, and carbonates, can also be used to ensure uniform Zn deposition stripping and inhibiting the occurrence of side reactions, such as Zn_3_(PO_4_)_2_·4H_2_O [83], Zn_3_(PO_4_)_2_ [84], Ca_5_(PO_4_)_3_(OH) [26], sepiolite (Si_12_O_30_Mg_8_(OH)_4_(H_2_O)_4_·8H_2_O) [85], kaolin (Al_2_Si_2_O_5_(OH)_4_) [86], and CaCO_3_ [87].

Recent studies indicate that phosphates tend to form layered structures with open skeletons, allowing the diffusion of protons in the interlayer region. This property provides good proton conductivity and electrocatalytic performance [88], promoting faster and more uniform deposition of Zn^2+^ on the Zn anode surface. In the meantime, this layer physically isolates the Zn anode from the electrolyte, thus suppressing dendritic growth and interfacial side reactions [89]. Li et al. [90] recently developed an ultra-thin (45 ± 5 nm) Zn phosphate protective layer on the Zn metal surface through a simple and rapid chemical treatment with polyphosphoric acid (PPA). This layer acts as an artificial Solid electrolyte interface that effectively prevents the corrosion reaction of the Zn anode, achieving uniform Zn deposition and stripping. The PPA-Zn electrode with the optimal protective layer thickness exhibits stable cycling for 6500 h at a current density of 2 mA cm^-2^.

**Fig. 8:**
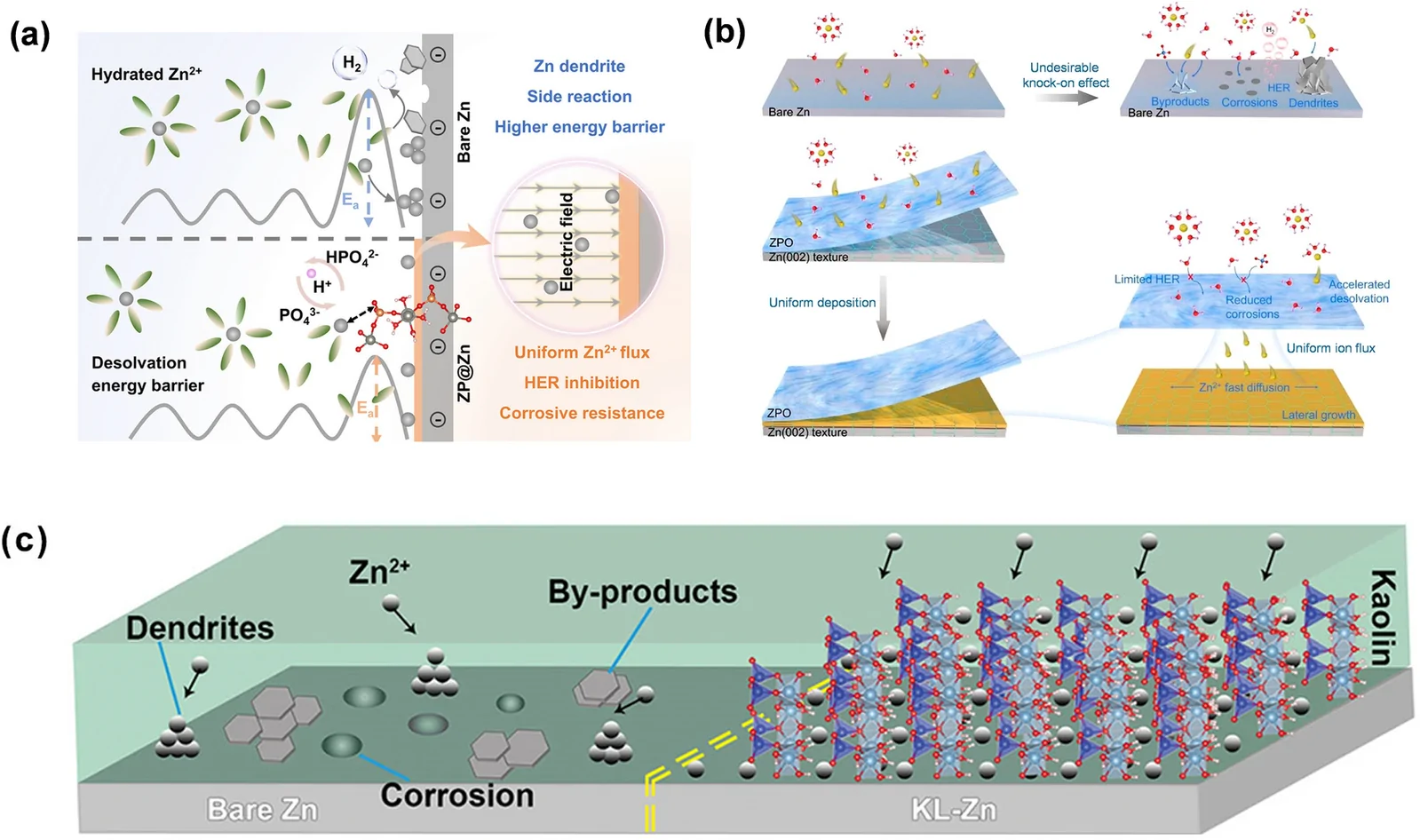
Zn deposition on different inorganic acid salt layers.** a** Schematic diagram of the Zn^2+^ deposition process on bare Zn and Zn_3_(PO_4_)_2_·4H_2_O@Zn [91]. Copyright 2022, Wiley‐VCH. **b** Schematic diagram of different galvanized layers on bare Zn and Zn(002)@Zn_3_(PO_4_)_2_ [84]. Copyright 2023, American Chemical Society. **c** Schematic diagram of the Zn^2+^ deposition process on bare Zn and KL-Zn anodes [86]. Copyright 2020, Wiley-VCH

Beyond their established role in regulating Zn ion diffusion in zinc ion batteries, phosphates have demonstrated additional mechanisms and applications. It has been revealed that phosphate coatings can buffer electrolyte pH changes, thereby suppressing HER [81, 91]. The authors developed a reversible proton storage SEI layer (Zn_3_(PO_4_)_2_·4H_2_O@Zn) on the Zn anode. During charging, the reversible conversion between Zn_3_(PO_4_)_2_·4H_2_O and ZnHPO_4_/Zn(H_2_PO_4_)_2_ competes with HER for protons. While during discharge, it releases these protons. Through this dual mechanism of HER regulation and Zn^2+^ diffusion control, the protective layer promotes uniform Zn deposition (Fig. 8a). More insight to the mechanism is provided by Song et al. [84] who looked specifically into the effect of the Zn crystal orientation and an in situ formed amorphous Zn_3_(PO_4_)_2_ protection layer (Fig. 8b). DFT calculations show that the Zn(002)@ZPO interface gives a Zn^2+^ diffusion energy barrier that is nearly one order of magnitude lower than those at Zn(100)@ZPO and Zn(101)@ZPO interfaces. This low diffusion energy barrier facilitates Zn^2+^ transport and homogeneous distribution, particularly at high rates. The Zn(002)@ZPO achieved a cycle Life exceeding 500 h in a symmetrical cell under a high areal capacity of 10 mAh cm^-2^ (DOD: 68%).

While inorganic coatings enable uniform zinc deposition through high Zn^2+^ conductivity, their brittle nature induces structural fracture during rapid plating/stripping cycles. Yang et al. [92] addressed this limitation by constructing an organic–inorganic NFZP (Nafion/Zn_3_(PO_4_)_2_) bilayer: A bottom Zn_3_(PO_4_)_2_ layer with high Zn^2+^ conductivity facilitates ion transport, while a top Nafion layer accommodates anode volume changes and blocks water penetration. The Nafion bridges the inorganic component to form a compact coating, synergistically suppressing dendrite growth and hydrogen evolution. This Dual-layer design achieves a 2,800 h cycling Lifespan with 45 mV overpotential under 0.5 mA cm⁻^2^ in symmetric cells. This approach of constructing an organic/inorganic SEI interface has also achieved promising results in both lithium-ion and sodium-ion batteries [93, 94].

Silicates have gained popularity among researchers due to their exceptional structural stability. Wang et al. [85] achieved precise control over water molecules in sepiolite (Si_12_O_30_Mg_8_(OH)_4_(H_2_O)_4_·8H_2_O) through heat treatment. The structural water (Mg-OH) in Sep-OH ensures layer stability, and the oxygen in Si-O-2Mg bonds and -OH groups exhibits strong adsorption capacity for Zn^2+^. This limits the lateral movement of Zn^2+^, effectively inhibiting the growth of Zn dendrites. Additionally, the Sep-OH layer demonstrates hydrophobicity toward free water molecules in the electrolyte, enhancing the corrosion resistance of Zn anode. Consequently, at a high current density of 5 mA cm^-2^, the Zn anode protected by sepiolite layers exhibits a long cycle Life of 800 h. Deng et al. [86] employed kaolin (Al_2_Si_2_O_5_(OH)_4_) as a protective layer for the anode in AZMBs. Within the nanochannels of kaolin, Zn ions can swiftly move under the influence of hydrogen–oxygen bonds, but they cannot cross between channels, effectively regulating the irregular two-dimensional diffusion of Zn ions (Fig. 8c). Leveraging kaolin's selectivity for elements, uniform-pore distribution, and abundant adsorption sites, the deposition process of Zn ions is guided. This protective layer demonstrates excellent electrochemical performance, dendrite suppression in both symmetrical and full-cell conditions.

Furthermore, vermiculite, as a type of silicate, has a small lattice mismatch of only 0.38% with Zn(002) [95]. A low lattice mismatch means small lattice strain of the deposits on the substrate, leading to an epitaxial interface and strong orientation correlation. For example, Zheng et al. [95] synthesized a porous vermiculites (MPVMTs) monolayer coating on Zn anodes (MPVMT@Zn). The MPVMT layer has a lattice constant double that of Zn, leading to Zn(002) nucleation and horizontal growth of Zn. Consequently, the modified symmetric cells operated stably for over 300 hours at a high current density of 50 mA cm⁻^2^. And even under the harsh conditions of high zinc utilization (51%, with a zinc thickness of 10 μm) and a low N/P ratio (Negative/Positive ratio) of 1.9, the MPVMT coatings achieve stable cycling of the zinc anodes and Maintain nearly 100% capacity after 200 cycles, which is more than four times better than the performance of bare Zn || I₂ cells.

Based on the various interface materials discussed above for zinc anode protection, we have summarized their corresponding battery performance metrics. As shown in Table 1, the implementation of protective materials consistently enhances battery cycle life by factors ranging from several to dozens, demonstrating the critical role of inorganic layers in zinc anode protection. However, direct comparisons between different materials face significant challenges due to variations in test parameters and battery assembly conditions. The diverse experimental parameters, particularly current density and areal capacity, complicate the assessment of battery performance levels. Moreover, the disparities in battery assembly conditions are evidenced by the inconsistent performance of unmodified zinc batteries (control groups) across different studies, which exhibit notable differences even under identical test parameters. These variations in control group performance further limit meaningful comparisons of experimental results across different studies.

**Table 1:** Inorganic interface engineering strategies for advanced Zn metal anodes

Inorganic coating materials	Current density (mA cm^-2^)	Area capacity (mAh cm^−2^)	Lifespan (hour)	Areal cumulative capacity (mAh cm^−2^)	Voltage hysteresis (mV)	Refs.
Al_2_O_3_	1	1	500	250	36.5	[63]
Sc_2_O_3_	1	1	200	100	48	[64]
Ti_4_O_7_	1	1	2600	1300	24.2	[96]
ZrO_2_	1	1	6000	3000	48	[97]
Nb_2_O_5_	0.25	0.125	1000	125	86	[65]
ZnO	5	1	1765	4412.5	50	[32]
ZnO-3D	5	1.25	500	1250	43	[62]
ZnO HPA	1	1	400	200	39.3	[61]
ZnVO	0.25	0.05	560	70	50	[67]
CrN	1	0.25	3700	1850	41	[69]
TiN	1	1	2300	1150		[70]
ZnS	17	17	1000	8500	86	[74]
ZnSe	1	1	1500	750	15.5	[75]
ZnSe	1	1	1530	765	32	[72]
MXene	0.2	0.2	800	80	47	[98]
Cu-MXene	10	1	1000	5000	120	[36]
Ti_3_C_2_Tx MXene	5	1	650	1625	80	[82]
Ti_3_C_2_Cl_2_ MXene	10	0.5	700	3500	65	[81]
Zincophilic MXene	2	2	3600	3600	60	[80]
ZP	5	1.25	2000	5000		[91]
Zn phosphate	2	1	6500	6500		[90]
Zn(002)@Zn_3_(PO_4_)_2_	20	10	500	5000		[84]
Hydroxyapatite	15	10	1200	9000		[26]
Ca_5_(PO_4_)_3_F	1	0.5	4000	2000	48	[99]
Sepiolite	5	1	800	2000	45	[85]
kaolin	4.4	1.1	800	1760	70	[86]

## Summary and Outlook

**Fig. 9:**
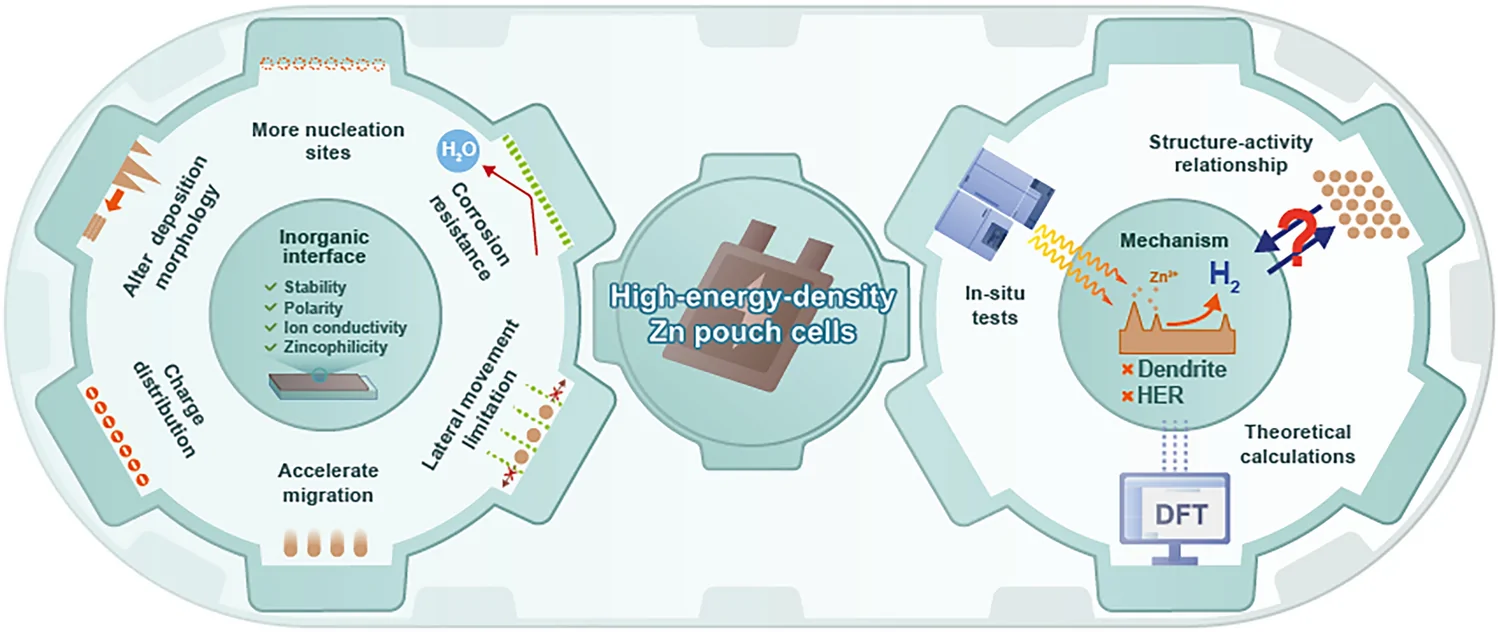
Summary and outlook of the inorganic interface engineering strategies for advanced Zn metal anodes

We have provided a comprehensive overview of inorganic interface engineering strategies for advanced Zn metal anodes, focusing on their mechanisms for regulating the Zn^2^⁺ plating/stripping process (Fig. 9). The high dielectric constants, polarity, and zincophilic sites of inorganic materials help to homogenize charge distribution at the plating interface and increase nucleation sites, promoting uniform Zn nucleation. Additionally, the high Zn^2^⁺ adsorption capacity and the vertical electric field between the Zn substrate and the inorganic protective layer enable fast, even migration of Zn^2+^ ions, supporting rapid and uniform crystal growth even at high current densities. Some inorganic layers can further regulate crystal planes and alter Zn deposition morphology, effectively reducing dendrite formation.

Despite significant advancements in AZMBs performance, there remains a gap between the current state and practical needs. A major challenge in designing high-performance Zn^2+^ plating/stripping interfaces is the unclear structure–activity relationship between interfacial layer composition/structure and electrochemical performance. This gap limits the establishment of clear standards and guiding principles for effective interface design.

(1) *Composition of Solid Electrolyte Interphase (SEI):* Inorganic coatings, despite their numerous advantages, predominantly employ rigid materials that exhibit a relatively weak physical coupling with the zinc substrate. This weak coupling makes them prone to detachment due to volume expansion during prolonged cycling, ultimately leading to coating failure. An ideal SEI film, either pre-deposited (i.e., artificial SEI) or by electrolyte decomposition, is a composite consisting of organic and inorganic phases. The organic phase can effectively eliminate water-related side reactions and enhance the flexibility of the coating, while the inorganic phase promotes rapid ion conduction and provides mechanical strength.

(2) *Systematic Theoretical Studies for Dendrite Suppression:* To better guide interface design for dendrite control, future studies should take a broader approach beyond individual materials, incorporating comprehensive analyses across diverse inorganic layers. In addition to deeper understanding of HER mechanism and process, integrating high-throughput calculations with experimental methods will help clarify how composition, structure, and interfacial properties (such as chemical bonding, lattice matching) of the interface layer influence Zn electroplating/stripping dynamics. This approach is essential for establishing key descriptors for rational design of materials. In addition, Zn ion conductivity and the corresponding transference number within this inorganic interface should also be properly evaluated.

(3) *Enhanced Understanding of HER Regulation:* The extended cycle life required for electrical energy storage (EES) demands highly reversible zinc plating and stripping. Corrosion and hydrogen evolution reaction (HER) on zinc anodes reduce Coulombic efficiency but appear less significant in high current and rate cycling tests. It is recommended that Coulombic efficiency should be also evaluated at low current densities to avoid pseudo-high values. It would be helpful to develop in situ methods to monitor the dynamics of hydrogen gas bubble evolution and detachment at the interfaces.

(4) *Toward High-Energy-Density Pouch Cells:* For Zn-based energy storage to be viable in real-world applications, high-energy-density pouch cells are the holy grail. Increased materials loading, choice of cathode materials, and lean or rich electrolyte condition (corresponding to different mass ratios with the active materials), as well as different assembly methods can significantly impact the cell performance. For example, Zn plating/stripping at high areal capacities may alter the interaction between the inorganic layer and Zn metal, affecting interface stability. Measuring at small rates may lead to several self-discharge. Differences in pressure between pouch cells and laboratory-scale coin cells also influence dendrite growth. Parameters like inorganic layer thickness, which impacts energy and power density, need be well balanced to avoid increased polarization and dendrite formation. Careful optimization of these design factors will be crucial for transitioning Zn anodes with inorganic interfaces to high-energy-density battery systems.

(5) *Testing Protocols:* To advance the development of AZMBs, it is crucial to establish protocols for battery assembly, electrolyte volume, and zinc anode usage. Standardizing the evaluation of zinc anodes will allow consistent testing conditions, enabling accurate comparisons across different studies and reducing discrepancies, for instance, the testing method of Coulombic efficiency. Standardized guidelines will enhance the reliability of electrochemical measurements and facilitate the transition from laboratory to industry. These efforts, along with inorganic interface engineering strategies, are expected to play a pivotal role in enabling practical, dendrite-free zinc metal batteries.
